# Is diagnosis enough to guide interventions in mental health? Using case formulation in clinical practice

**DOI:** 10.1186/1741-7015-10-111

**Published:** 2012-09-27

**Authors:** Craig A Macneil, Melissa K Hasty, Philippe Conus, Michael Berk

**Affiliations:** 1 Early Psychosis Prevention & Intervention Centre (EPPIC), and Orygen Youth Health Research Centre, Orygen Youth Health, 35 Poplar Road, Parkville, Melbourne, Victoria, 3052, Australia; 2 Orygen Research Centre, 35 Poplar Road, Parkville, Victoria, 3052, Australia; 3 Treatment & Early Intervention in Psychosis Program (TIPP), Département de Psychiatrie CHUV, Université de Lausanne, Clinique de Cery, 1008 Prilly, Switzerland; 4 School of Medicine, Deakin University, 1 Gheringhap Street, Geelong, Victoria, 3220, Australia; 5 Department of Psychiatry, The University of Melbourne, Level 1 North, Main Block, Royal Melbourne Hospital, Victoria, 3050, Australia; 6 Mental Health Research Institute, Kenneth Myer Building, 30 Royal Parade, Parkville Victoria, 3052, Australia

**Keywords:** Formulation, diagnosis, cognitive behavioral therapy, psychological intervention, case conceptualization

## Abstract

While diagnosis has traditionally been viewed as an essential concept in medicine, particularly when selecting treatments, we suggest that the use of diagnosis alone may be limited, particularly within mental health. The concept of clinical case formulation advocates for collaboratively working with patients to identify idiosyncratic aspects of their presentation and select interventions on this basis. Identifying individualized contributing factors, and how these could influence the person's presentation, in addition to attending to personal strengths, may allow the clinician a deeper understanding of a patient, result in a more personalized treatment approach, and potentially provide a better clinical outcome.

## Background

*"In neurosis and personality disorders, formulation is a clearer guide to aetiology, prognosis and treatment than is categorical diagnosis" *Aveline, p. 199 [1]

This paper describes concerns around using diagnosis alone as a tool to select clinical interventions, provides an overview of some current models of case formulation, and examines its potential clinical utility.

## The clinical utility of diagnosis and formulation

With debate and controversy already emerging around some of the diagnoses proposed in the upcoming *Diagnostic and Statistical Manual of Mental Disorders *(DSM), Fifth Edition, it may be timely to review the concepts of diagnosis and formulation in mental health. Psychiatry has traditionally emphasized the importance of diagnostic categories, with the implication that these offer a reliable guide for treatment options and prediction of outcomes. Diagnosis has also been regarded historically as helpful from a research standpoint, allowing categorization of people by disorders in order to quantify outcomes, and facilitate discussion around interventions and etiology.

In reality, however, diagnosis alone may tell us little about causation of a psychiatric disorder. Diagnosis may also instruct us poorly about which form of intervention we should undertake, and offers no information about the person's experience of their disorder. Kendell and Jablensky [2] acknowledged that while diagnoses may be '...helpful working concepts for clinicians' (p.4), many are not 'valid', in the sense that they are not '...discrete entities with natural boundaries that separate them from other disorders' (p.4). Furthermore, Tarrier and Calam [3] noted that, as diagnoses in the DSM and the *International Classification of Diseases *(ICD-10) are often based on selecting from a list in which some items are present and others absent, it is possible for two people to have the same diagnosis with few, and in some cases, no symptoms in common.

Categorical diagnoses are perhaps most valuable for disorders in which there is greater homogeneity, where biomarker studies show some demonstrable patterns, and where categorical diagnosis guides treatment with a degree of accuracy. Unfortunately, few disorders in psychiatry match this description. Phenomena such as mood and personality disorders, psychoses, and anxiety disorders can be associated with a diversity of etiological factors including early childhood experiences, trauma, personality styles, family, interpersonal, lifestyle, medical, and social stressors, with each factor playing a greater or lesser role for each person. Understanding and incorporating these into an individualized treatment plan is an essential part of quality care, with failure to do so not only risking an ineffective outcome, but potentially impacting negatively on the therapeutic relationship and resulting in exacerbation of the person's symptomatology.

At least in part due to recognition of the limitations of diagnosis in mental health, the concept of formulation or case conceptualization has attracted increasing interest in recent years. Formulation has been defined as synthesizing the patient's experience with relevant clinical theory and research [4], as '...the bridge between assessment and treatment' [[5], p.210] and has been utilized for multiple disorders in children, adults and older adults. [6-8]. However, formulation may be particularly helpful for people who have not had an adequate response to traditional interventions, people who have Axis II disorders, or when comorbidity complicates which interventions should be utilized first [9,10].

Formulation can serve a number of functions. These include: understanding significant etiological factors that have influenced the person's presentation; identifying key difficulties; guiding which interventions should be utilized and in what order; and anticipating challenges that may occur during the course of treatment [4,6,7,11].

## What should a formulation comprise? The 'Five P's' approach to formulation

Despite some differences between theoretical orientations, some key themes exist around the content of formulations, with one of the more popular recent approaches utilizing the 'Five Ps'. These are:

1. Presenting problem. This goes beyond diagnosis to include what the person and clinician identify as difficulties, how the person's life is affected, and when a particular difficulty should be targeted for intervention. For example, while a person may meet criteria for the diagnosis of borderline personality disorder, presenting difficulties may include not being able to maintain employment, erratic friendships, and physical health complications resulting from self-harm. Specifying such difficulties can allow for a more focused intervention.

2. Predisposing factors. This comprises identifying possible biological contributors (for example, organic brain injury and birth difficulties), genetic vulnerabilities (including family history of mental health difficulties), environmental factors (such as socio-economic status, trauma, or attachment history) and psychological or personality factors (including core beliefs or personality factors) which may put a person at risk of developing a specific mental health difficulty.

3. Precipitating factors. This can include significant events preceding the onset of the disorder, such as substance use, or interpersonal, legal, occupational, physical, or financial stressors.

4. Perpetuating factors. This comprises factors which maintain the current difficulties. These can include ongoing substance use, repeating behavioral patterns (including avoidance or safety behaviors in anxiety disorders, or withdrawal in depressive disorders), biological patterns (such as insomnia in mania, and insomnia or hypersomnia in depression) or cognitive patterns such as attentional biases, memory biases, or hypervigilance.

5. Protective/positive factors. This involves identifying strengths or supports that may mitigate the impact of the disorder. These can include social support, skills, interests, and some personal characteristics. Kuyken *et al*. [4] suggested that this is a particularly important element which has traditionally been lacking in mental health interventions, but inclusion of which results in a higher likelihood of reduced symptomatology and increased resilience (p.4). We would add that identification of protective factors also creates increased optimism in both the clinician and patient and contributes to a positive therapeutic relationship.

Importantly, formulations should be flexible, and should incorporate new information as it emerges. As Persons [6] noted, '... assessment and treatment are a continuous process of proposing, testing, re-evaluating, revising, rejecting, and creating new formulations' (p. 55).

## Cautions Regarding Formulation

Despite numerous strengths, some caution is required when using a formulation-based approach. Chadwick *et al*. [12] reported that while most of their participants reported an '...increased sense of hope and understanding' (p. 679) following formulation, some also described negative aspects, including that it has been 'saddening, upsetting and worrying' (p. 674). This study highlights the importance of developing collaborative formulations within a strong therapeutic relationship and at a reasonable pace. We would also suggest that similar risks exist when discussing diagnoses with patients. It is also notable that the formulation undertaken in this study did not include identifying participants' strengths or protective factors. It may be, therefore, that had protective factors and strengths been included, the outcome could have been better.

Notably, research on the impact of formulation on outcome is still somewhat limited, and questions remain regarding inter-rater reliability [4,13]. However, training and therapist experience have consistently been found to impact on the quality of formulations for clinicians working with either cognitive behavioral or psychodynamic frameworks [14]. Encouragingly, Kendjelic & Eells [15] reported that even brief clinician training produced '... formulations rated as higher in overall quality and as more elaborated, comprehensive, complex, and precise' (p.66).

## Conclusions

When done well, formulation provides an opportunity for a shared understanding of a person's difficulties, and can offer a way of answering the classic questions of 'why this person?', 'why this problem?', and 'why now?' in ways that diagnosis alone does not. Importantly, it can also provide a rationale and shared agenda for what to target and in what order. While there may be some risks involved in formulation, if done sensitively, collaboratively, and accounting for strengths, we suggest it can be a clinical tool with the potential to provide considerably better outcomes than diagnosis alone. A challenge is the integration of a formulation-based management plan with categorical diagnosis and the current evidence base to provide a broad-based clinical understanding and an individualized therapeutic strategy.

## Competing interests

CAM has received past honoraria and travel grants from AstraZeneca, Eli Lilly, Sanofi-Aventis and Janssen-Cilag for speaking engagements and attendance of advisory committees. MKH has received a travel grant from Pfizer and research funding from AstraZeneca. PC has received research grants and travel grants to attend conferences from Bristol Meyers Squibb, Astra Zeneca, Jannsen Cilag, and Eli Lilly. MB has received Grant/Research Support from the NIH, Cooperative Research Centre, Simons Autism Foundation, Cancer Council of Victoria, Stanley Medical Research Foundation, MBF, NHMRC, Beyond Blue, Geelong Medical Research Foundation, Bristol Myers Squibb, Eli Lilly, Glaxo SmithKline, Organon, Novartis, Mayne Pharma and Servier, has been a speaker for Astra Zeneca, Bristol Myers Squibb, Eli Lilly, Glaxo SmithKline, Janssen Cilag, Lundbeck, Merck, Pfizer, Sanofi Synthelabo, Servier, Solvayand Wyeth, and served as a consultant to Astra Zeneca, Bristol Myers Squibb, Eli Lilly, Glaxo SmithKline, Janssen Cilag, Lundbeck and Servier. None of the above companies were involved in any way in the drafting, reviewing or in providing financial assistance for this paper.

## Authors' contributions

CAM and MKH were involved in the conceptual background and drafting the manuscript. PC and MB were involved in critically revising the paper.

## Pre-publication history

The pre-publication history for this paper can be accessed here:

http://www.biomedcentral.com/1741-7015/10/111/prepub
